# Use of Outpatient Cardiac Rehabilitation Among Heart Attack Survivors — 20 States and the District of Columbia, 2013 and Four States, 2015

**DOI:** 10.15585/mmwr.mm6633a1

**Published:** 2017-08-25

**Authors:** Jing Fang, Carma Ayala, Cecily Luncheon, Matthew Ritchey, Fleetwood Loustalot

**Affiliations:** 1 Division for Heart Disease and Stroke Prevention, National Center for Chronic Disease Prevention and Health Promotion, CDC.

Heart disease is the leading cause of death in the United States (*1*). Each year, approximately 790,000 adults have a myocardial infarction (heart attack), including 210,000 that are recurrent heart attacks (*2*). Cardiac rehabilitation (rehab) includes exercise counseling and training, education for heart-healthy living, and counseling to reduce stress. Cardiac rehab provides patients with education regarding the causes of heart attacks and tools to initiate positive behavior change, and extends patients’ medical management after a heart attack to prevent future negative sequelae (*3*). A systematic review has shown that after a heart attack, patients using cardiac rehab were 53% (95% confidence interval [CI] = 41%–62%) less likely to die from any cause and 57% (95% CI = 21%–77%) less likely to experience cardiac-related mortality than were those who did not use cardiac rehab (*3*). However, even with long-standing national recommendations encouraging use of cardiac rehab (*4*), the intervention has been underutilized. An analysis of 2005 Behavioral Risk Factor Surveillance System (BRFSS) data found that only 34.7% of adults who reported a history of a heart attack also reported subsequent use of cardiac rehab (*5*). To update these estimates, CDC used the most recent BRFSS data from 2013 and 2015 to assess the use of cardiac rehab among adults following a heart attack. Overall use of cardiac rehab was 33.7% in 20 states and the District of Columbia (DC) in 2013 and 35.5% in four states in 2015. Cardiac rehab use was underutilized overall and differences were evident by sex, age, race/ethnicity, level of education, cardiovascular risk status, and by state. Increasing use of cardiac rehab after a heart attack should be encouraged by health systems and supported by the public health community.

The BRFSS is a telephone survey, conducted annually by all U.S states, with guidance and support from CDC (https://www.cdc.gov/brfss). The survey includes a core component and optional modules. Participants with history of a heart attack are identified by an affirmative response to the question, “Has a doctor, nurse, or other health professional ever told you that you had a heart attack, also called a myocardial infarction?” In 2013, 20 states* and DC, and in 2015, four states^†^ included the cardiovascular health module, which contained questions about using cardiac rehab after a heart attack. The median response rates for the BRFSS were 46.4% and 47.2% for 2013 and 2015, respectively.

Participants identified as heart attack survivors were asked: “After you left the hospital following your heart attack, did you go to any kind of outpatient rehabilitation?” Demographic characteristics included age, sex, race/ethnicity (non-Hispanic white, non-Hispanic black, non-Hispanic other, or Hispanic), highest level of education achieved (less than high school, high school graduate, some college, or college graduate) and having any kind of health insurance. Selected self-reported cardiovascular disease (CVD) risk factors included hypertension, high blood cholesterol, diabetes, obesity, and current smoking.^§^ Each respondent was categorized based on their number of CVD risk factors (0, 1, 2, 3, 4, or 5). Among heart attack survivors, the crude and adjusted percentage of cardiac rehab use was assessed overall and by state of residence in 2013 and 2015, as well as by demographic characteristics and CVD risk in 2013. P-values were obtained by Wald F test and p<0.05 were used to identify statistically significant differences among subgroups. The BRFSS’s complex sample design was accounted for using statistical software with BRFSS respondent sampling weights and design variables.

In 2013, a total of 166,913 participants who completed the cardiovascular health module from 20 states and DC, among whom, 4.8% (95% CI = 4.6–5.0) were heart attack survivors. In 2015, a total of 20,776 participants from four states completed the module, 4.3% (3.9–4.7) of whom were heart attack survivors. Overall, 33.7% (95% CI = 31.8–35.6) of heart attack survivors in 2013 and 35.5% (95% CI = 31.0–40.3) in 2015 reported use of cardiac rehab after leaving the hospital following their heart attack.

In 2013, among 9,490 heart attack survivors, older adults, men, non-Hispanic whites, persons with college or higher education, and those with two, three, or four (of five) CVD risk factors were more likely to receive cardiac rehab than were younger persons, women, non-Hispanic blacks, Hispanics, persons with less than a college education, and persons with fewer than two or with five out of five CVD risk factors (relative to those with two, three of four; p<0.05) (Table 1).

**TABLE 1:** Crude and adjusted percentages* of adults who survived a heart attack and received cardiac rehabilitation, by descriptive characteristics — Behavioral Risk Factor Surveillance System, 20 U.S. states and the District of Columbia, 2013

Characteristics	No.	Crude % (95% CI)	p-value	Adjusted %* (95% CI)	p-value
**Total**	**9,490**	**33.7 (31.8–35.6)**	**<0.001**	**33.7 (31.8–35.6)**	**<0.001**
**Sex**			<0.001	—	<0.001
Men	5,197	36.9 (34.4–39.5)		36.4 (33.9–39.0)	
Women	4,293	28.2 (25.7–30.8)		28.8 (26.4–31.4)	
**Age group (yrs)**			<0.001	—	<0.001
18–64	3,197	26.9 (24.3–29.7)		28.6 (26.0–31.3)	
≥65	6,293	39.6 (37.2–42.1)		37.9 (35.3–40.5)	
**Race/Ethnicity**			<0.001	—	<0.001
White, non-Hispanic	7,756	37.0 (35.0–38.9)		35.4 (33.5–37.4)	
Black, non-Hispanic	873	21.9 (17.4–27.3)		25.3 (20.4–31.0)	
Hispanic	617	23.2 (17.5–30.0)		24.5 (18.4–31.8)	
Other, non-Hispanic	244	25.7 (15.9–38.8)		33.3 (22.4–46.3)	
**Education**			<0.001	—	<0.001
Less than high school	1,483	21.8 (17.7–26.6)		23.3 (19.4–27.6)	
High school diploma	3,297	36.2 (33.3–39.3)		36.1 (33.1–39.2)	
Some college	2,649	33.6 (30.5–36.8)		33.0 (29.9–36.2)	
College graduate	2,061	48.3 (44.1–52.4)		46.4 (42.5–50.4)	
**Insurance**			<0.001	—	0.0197
Yes	8,899	35.3 (33.4–37.3)		34.4 (32.5–36.5)	
No	591	18.6 (13.7–24.8)		25.2 (19.0–32.5)	
**No. of cardiovascular risk factors^†^**			0.0108	—	0.0074
0	557	32.3 (25.5–40.0)		32.3 (25.8–39.6)	
1	1,719	27.9 (23.8–32.4)		27.2 (23.5–31.3)	
2	2,987	36.7 (33.1–40.4)		35.4 (32.0–39.0)	
3	2,671	35.2 (32.0–38.5)		35.1 (32.1–38.2)	
4	1,380	34.1 (29.6–38.8)		37.1 (32.8–41.7)	
5	176	21.3 (13.6–31.8)		25.7 (16.8–37.2)	

**Abbreviation:** CI = confidence interval.

*Adjusted for age, sex, race/ethnicity, education, insurance status, and CVD risk.

**^†^** Hypertension, high cholesterol, diabetes, obesity, and current smoker.

In 2013, the adjusted percentage of cardiac rehab use ranged from 20.7% in Hawaii to 58.6% in Minnesota (Table 2). Among the four states that used the cardiac rehab module in 2015, both the crude and adjusted percentages of cardiac rehab use were lowest in Georgia and highest in Iowa. Among the four states that used the module in both 2013 and 2015, the overall adjusted percentage of cardiac rehab use was 35.6% (95% CI = 32.1–39.3) in 2013 and 35.5% (95% CI = 31.0–40.3) in 2015 (p = 0.8075).

**TABLE 2:** Number and crude and adjusted percentages* of adults who survived a heart attack and received cardiac rehabilitation, by state — Behavioral Risk Factor Surveillance System, 20 U.S. states and the District of Columbia (DC) (2013) and 4 U.S. states (2015)

States^†^	No.	Crude		Adjusted*	
		% (95% CI)	p-value	% (95% CI)	p-value
**2013 (20 states and DC)**			<0.001	—	<0.001
**Total**	**9,490**	**33.7 (31.8–35.6)**		**33.7 (31.8–35.6)**	
Hawaii	263	19.7 (13.6–27.8)		20.7 (13.9–29.6)	
Oklahoma	288	20.8 (15.7–27.0)		20.9 (15.6–27.2)	
Oregon	225	26.9 (20.5–34.4)		24.9 (19.2–31.7)	
Arizona	230	23.5 (15.1–34.6)		25.0 (17.7–34.2)	
Tennessee	392	25.0 (19.9–30.9)		27.2 (21.9–33.2)	
Washington	550	31.2 (26.3–36.5)		29.4 (24.8–34.5)	
DC	183	23.6 (16.1–33.2)		29.5 (20.0–41.1)	
Mississippi	458	27.8 (21.9–34.6)		29.5 (23.6–36.3)	
Florida	2,288	30.4 (25.7–35.5)		29.9 (25.8–34.4)	
Georgia	375	28.6 (23.2–35.1)		30.1 (24.5–36.3)	
North Carolina	227	29.1 (22.3–37.0)		31.2 (24.3–39.0)	
Arkansas	345	30.0 (23.6–37.3)		31.5 (25.0–38.9)	
Missouri	470	36.6 (30.3–43.4)		36.3 (30.1–43.0)	
South Carolina	569	37.7 (32.4–43.3)		38.3 (33.1–43.8)	
Massachusetts	195	46.5 (36.0–57.4)		42.9 (33.4–53.0)	
Maine	286	48.6 (41.3–56.0)		46.1 (38.7–53.7)	
North Dakota	392	51.7 (45.3–58.0)		47.2 (41.1–53.3)	
Nebraska	456	51.4 (44.2–58.5)		49.0 (42.3–55.8)	
Iowa	464	54.6 (48.9–60.2)		51.4 (45.7–57.0)	
Wisconsin	266	56.3 (45.9–66.1)		53.3 (44.0–62.4)	
Minnesota	568	60.9(52.4–68.8)		58.6 (49.9–66.7)	
**2015 (four states)**			<0.001	—	<0.001
**Total**	**1,006**	**35.5 (31.0–40.3)**		**35.5 (31.0–40.3)**	
Georgia	229	26.3 (19.9–34.0)		27.9 (21.5–35.5)	
Oregon	206	35.5 (27.6–44.3)		32.2 (24.6–40.9)	
Maine	294	45.0 (37.8–52.4)		44.4 (36.9–52.1)	
Iowa	277	59.4 (52.0–66.5)		57.5(49.6–65.0)	

**Abbreviation:** CI = confidence interval.

*Adjusted for age, sex, race/ethnicity, education, insurance status and CVD risk.

^†^ States are listed in ascending order of adjusted percentage of receiving cardiac rehabilitation in 2013 and 2015.

## Discussion

In this analysis, approximately 1 in 3 heart attack survivors reported receiving cardiac rehab after suffering a heart attack. These estimates highlight missed opportunities to access an evidenced-based intervention that has been documented to improve patient survival, quality of life, functional status, and cardiovascular risk profile following a significant health event, as well as reduce risk for a recurrent heart attack and psychological disorders (*3*,*6*).

Outpatient cardiac rehab has historically been underutilized (*5*), and the findings from this report demonstrate that this continues to be the case in all groups. No subgroup examined had utilization rates exceeding 50% and no state had utilization rates above 61%. Even with low percentages of rehab use, disparities in its use were apparent. Younger adults, females, blacks, Hispanics, adults without health insurance, and those with fewer than two or with five out of five CVD risk factors (relative to those with two, three, or four) were less likely to use cardiac rehab than were their counterparts. Threefold differences in cardiac rehab use were observed at the state level. The continued underutilization of cardiac rehab overall and among the aforementioned subgroups has been shown to be related to multiple factors, including lack of patient knowledge, awareness, and perceived importance of rehab; accessibility to rehab program sites; lack of health insurance coverage or high out-of-pocket costs for these services; and low referral rates from health care professionals (*4*).

In concert with *Healthy People 2020* objectives (*7*), the U.S. Department of Health and Human Service’s Million Hearts initiative (https://millionhearts.hhs.gov/index.html), aims to increase cardiac rehab use among heart attack survivors across the United States (*8*). The Million Hearts Cardiac Rehabilitation Collaborative,^¶^ a group of over 30 organizations and agencies, has developed an action plan to increase use of cardiac rehab to over 70%. The roadmap for this action plan includes interventions that increase the referral to cardiac rehab (e.g., through electronic medical record-based referral), enrollment in rehab (e.g., via patient interaction with a cardiac rehab staff member liaison at hospital discharge), and adherence to cardiac rehab services (e.g., by minimizing patient copayments). Meeting the Million Hearts goal of increasing use of cardiac rehab among patients with a qualifying condition to ≥70% in 5 years would save an estimated 25,000 lives and prevent 180,000 hospitalizations annually in the United States (*9*).

The findings in this report are subject to at least four limitations. First, BRFSS data are self-reported and are limited by recall bias, which could lead to underestimation of either heart attacks or use of cardiac rehab. Second, the survey does not provide information about why survey respondents did not participate in cardiac rehab, or whether those who did had attended the recommended number of cardiac rehab sessions. Third, since state participation in using the rehab module of the BRFSS was low (40% in 2013 and 8% in 2015) and inconsistent over time, these findings do not provide nationally representative estimates. Finally, with relatively few respondents reporting a history of heart attack (183 [DC] to 2,288 [Florida]), state-level confidence intervals were wide and might account for nonsignificant differences in cardiac rehab use for some characteristics.

Health system interventions to promote cardiac rehab referral and use, supported by access to affordable rehab programs within the community, should be prioritized to improve outcomes and prevent recurrent events. Given that overall cardiac rehab use was low, improvement in referral is needed; however, populations with lower use of cardiac rehab, such as women, those with lower levels of education, and minority populations should be further assessed to determine barriers to the use of cardiac rehab. Some strategies that might improve use of cardiac rehab include higher payment for rehab by insurers, eliminating or reducing copays for patients, extending cardiac rehab clinic hours to improve access, as well as providing standardized referrals coupled with linkage to cardiac rehab staff member liaisons at hospital discharge or by primary care providers and cardiologists. In addition, patients who have experienced a heart attack should be made aware of the availability of alternative models of cardiac rehab, such as telehealth and home-based rehab, to reduce the barriers related to transportation and responsibilities at home or work (*4*,*6*,*9*,*10*).

**Summary**

**What is already known about this topic?**

Each year, approximately 210,000 heart attacks are recurrent events. Outpatient cardiac rehabilitation among heart attack survivors helps to reduce these recurrences and improve health outcomes. Thus, national guidelines and recommendations encourage the use of cardiac rehabilitation.

**What is added by this report?**

This report used the most recent Behavioral Risk Factor Surveillance System data from 2013 (20 states) and 2015 (four states) to assess the use of cardiac rehabilitation among adults following a heart attack. In 2013, only one third of heart attack survivors used cardiac rehabilitation, and its use varied by sex, race/ethnicity, education, insurance status, cardiovascular risk status and by state. The percentage of use of cardiac rehabilitation did not change significantly from 2013 to 2015 among the four states observed during both years.

**What are the implications for public health practice?**

The percentage of heart attack survivors using cardiac rehabilitation is suboptimal. Strategies that increase the use of cardiac rehabilitation among all heart attack survivors, including lowering out-of-pocket payment, improving access, standardizing referrals, and providing education to enhance awareness, with special focus among populations who are most underserved, has the potential to substantially improve health outcomes of heart attack survivors.

## Footnotes

* Arizona, Arkansas, Florida, Georgia, Hawaii, Iowa, Maine, Massachusetts, Minnesota, Mississippi, Missouri, Nebraska, North Carolina, North Dakota, Oklahoma, Oregon, South Carolina, Tennessee, Washington, and Wisconsin.

† Georgia, Iowa, Maine, and Oregon.

§ Hypertension was defined by answering “yes” to the question, “Have you ever been told by a doctor, nurse, or other health professional that you have high blood pressure?” (persons who answered yes only during pregnancy were not included); high blood cholesterol was defined by answering “yes” to the question, “Have you ever been told by a doctor, nurse, or other health professional that your blood cholesterol is high?”; diabetes was defined by answering “yes” to the question, “Have you ever been told by a doctor that you have diabetes?”; obesity was ascertained by asking, “About how much do you weigh without shoes?” and “About how tall are you without shoes?,” and based on the answers, calculating body mass index (kg/m2); obesity was defined as body mass index ≥30; current smoking was defined by answering “every day” or “some days” to the question, “Do you now smoke cigarettes every day, some days, or not at all?”

¶ The Million Hearts Cardiac Rehabilitation Collaborative (CRC), is an outgrowth of the Million Hearts Cardiac Rehabilitation Leadership Summit held in November 2015 in Washington, DC, with representatives from over 30 organizations and agencies as well as CR graduates and their families. Represented organizations include the American Association of Cardiovascular and Pulmonary Rehabilitation, American Heart Association, American College of Cardiology, American Association of Nurse Practitioners, American College of Physicians, American Hospital Association, Heart Failure Society of America, Preventive Cardiovascular Nurses Association, Blue Cross Blue Shield Association, National Medical Association, Patient-Centered Outcomes Research Institute, America’s Essential Hospitals, Mended Hearts, WomenHeart, and Visiting Nurse Services of NY, and MedStar Health. The CRC has grown to include additional clinical specialist and patient advocacy groups as well as representatives from CR programs and health systems across the country. The CRC meets quarterly by phone to drive progress on their aim of achieving at least 70% participation among those eligible by 2022.
